# A comparison of early versus late initiation of renal replacement therapy for acute kidney injury in critically ill patients: an updated systematic review and meta-analysis of randomized controlled trials

**DOI:** 10.1186/s12882-017-0667-6

**Published:** 2017-08-07

**Authors:** Xiao-mei Yang, Guo-wei Tu, Ji-li Zheng, Bo Shen, Guo-guang Ma, Guang-wei Hao, Jian Gao, Zhe Luo

**Affiliations:** 1 Department of Critical Care Medicine, Zhongshan Hospital, Fudan University, Shanghai, 200032 People’s Republic of China; 2 Department of Nursing, Zhongshan Hospital, Fudan University, Shanghai, People’s Republic of China; 3 Department of Nephrology, Zhongshan Hospital, Fudan University, Shanghai, People’s Republic of China; 4 Department of Nutrition, Zhongshan Hospital, Fudan University, Shanghai, 200032 People’s Republic of China; 5 Center of Clinical Epidemiology and Evidence-based Medicine, Fudan University, Shanghai, People’s Republic of China

**Keywords:** Renal replacement therapy, Timing, Acute kidney injury

## Abstract

**Background:**

To investigate the impact of timing the initiation of renal replacement therapy (RRT) on clinical outcomes in critically ill patients with acute kidney injury (AKI), focusing on the randomized controlled trials (RCTs) in this field.

**Methods:**

The PubMed, EMBASE and Cochrane databases were searched between January 1, 1985, and June 30, 2016, to identify randomized trials that assessed the timing of initiation of RRT in patients with AKI.

**Results:**

Nine RCTs, with a total of 1636 patients, were enrolled in this meta-analysis. A pooled analysis of the studies indicated no mortality benefit with “early” RRT, with an RR of 0.98 (95% CI 0.78 to 1.23, *P* = 0.84). There was no significant difference in intensive care unit (ICU) length of stay (LOS) or hospital LOS between the early and late RRT groups for survivors or nonsurvivors. Pooled analysis also demonstrated no significant change in renal function recovery (RR 1.02, 95% CI 0.88 to 1.19, I2 = 59%), RRT dependence (RR 0.76, 95% CI 0.42 to 1.37, I2 = 0%), duration of RRT (Mean difference 1.43, 95% CI -1.75 to 4.61, I2 = 78%), renal recovery time (Mean difference 0.73, 95% CI -2.09 to 3.56, I2 = 70%) or mechanical ventilation time (Mean difference − 0.95, 95% CI -3.54 to 1.64, I2 = 64%) between the early and late RRT groups. We found no significant differences in complications between the groups.

**Conclusions:**

Our meta-analysis revealed that the “early” initiation of RRT in critically ill patients did not result in reduced mortality. Pooled analysis of secondary outcomes also showed no significant difference between the early and late RRT groups. More well-designed and large-scale trials are expected to confirm the result of this meta-analysis.

**Electronic supplementary material:**

The online version of this article (doi:10.1186/s12882-017-0667-6) contains supplementary material, which is available to authorized users.

## Background

Acute kidney injury (AKI) is a common complication of critical illness that carries high morbidity and mortality rates. Among patients with AKI, approximately 20% require renal replacement therapy (RRT) [1, 2]. Apart from the modality, dialysis dose and anticoagulation, the optimal time to start RRT is considered an important determinant of the outcome of critically ill patients receiving RRT [3].

There are huge variations in the timing of RRT initiation in critically ill patients in the real world, based on the data from large randomized controlled trials (RCTs) [4, 5]. Although the early initiation of RRT was reported to be beneficial in critically ill patients with AKI [6, 7], this might expose patients to unnecessary RRT. Several meta-analyses have been published regarding the optimal timing of RRT initiation that achieved conflicting conclusions [8–11]. The paucity of RCTs involved in the meta-analysis precluded the establishment of definitive conclusions because non-RCTs may exaggerate the magnitude of the effect due to intrinsic and external factors. Moreover, the secondary outcomes, including renal function recovery, RRT dependence and mechanical ventilation time, have not been carefully studied in previous meta-analyses.

Recently, two well-designed RCTs were issued to evaluate the outcome of different strategies for RRT [12, 13]. The Artificial Kidney Initiation in Kidney Injury (AKIKI) trial demonstrated that mortality at 60 days was comparable between the groups (48.5% in the early-strategy group and 49.7% in the delayed-strategy group) [12]. In contrast, the ELAIN randomized clinical trial revealed that the early initiation of RRT significantly reduced the 90-day mortality compared with the delayed initiation of RRT [13]. These findings added further uncertainty about the efficacy of “early” RRT in critically ill patients. Despite numerous low-quality studies in this field, a definitive conclusion is still yet to be elucidated. Therefore, we aimed to conduct an updated systematic review and meta-analysis to evaluate the efficacy and safety of “early” initiation of RRT compared to “late” RRT in critically ill patients by collecting data from RCTs only because these represent the highest standard of evidence to support the optimal timing of RRT initiation.

## Methods

### Search strategy

We screened PubMed, EMBASE and Cochrane databases between January 1, 1985, and June 30, 2016, to identify randomized trials that assessed the timing of initiation of RRT in patients with AKI. The search strategies were restricted to human RCTs and English language. Studies published before 1985 were excluded because there has been great progress in RRT technology and critical care practices in recent years.

**Fig. 1:**
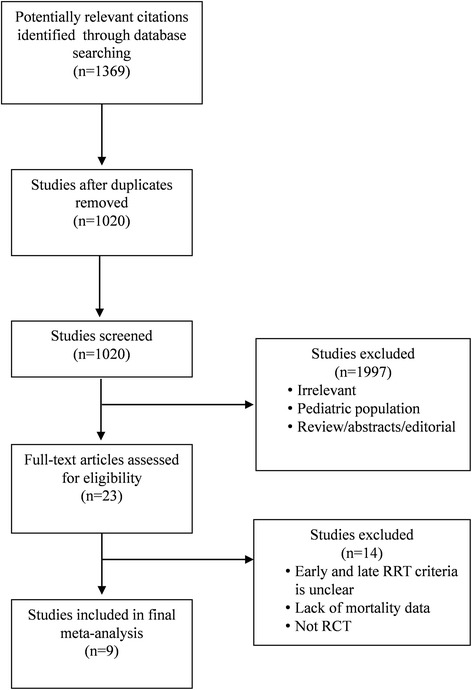
Flow chart of literature selection

The following keywords or medical subject headings were used: “acute kidney injury”, “acute kidney”, “acute renal”, “renal replacement therapy”, “renal replacement”, “hemodialysis”, “hemofiltration”, “dialysis”, “dialyzed”, “dialyzing”, “time to treatment”, “time”, “timing”, “initiation”, “start”, “accelerate”, “accelerated”, “accelerating”, “acceleration”, “early”, “earlier”, “late”. The search was slightly adjusted according to the requirements of the different databases. The authors’ personal files and reference lists of relevant review articles were also reviewed. The flow chart of the search strategies is summarized in Fig. 1.

### Study selection

Studies reporting the timing of RRT initiation in patients with AKI were selected for further review. The inclusion criteria were as follows: (1) randomized controlled trials; (2) adult critically ill population; and (3) clearly comparing early versus late RRT initiation with effect on mortality and/or clinically relevant secondary outcomes. We excluded studies without clear comparisons of the outcomes. A cursory review of titles and abstracts was independently performed by two reviewers (Xiao-mei Yang and Guo-wei Tu). Disagreement on the inclusion/exclusion of RCTs was resolved through consensus and, if necessary, consultation with a senior investigator (Zhe Luo). Retained RCTs were reviewed in detail.

### Data extraction

Data extracted included basic characteristics (author’s name, publication year, country of study, study period, study design, patient population, duration of follow-up, total number of patients, mean age, percentage of male), definition of “early” and “late” RRT, main characteristics at the time of RRT initiation (modality, creatinine, urine output, acute physiology and chronic health evaluation [APACHE II] score, sequential organ failure assessment [SOFA] score). The primary outcome was mortality, including 14-day mortality, 28-day mortality, 30-day mortality, 60-day mortality, 90-day mortality, ICU mortality, and in-hospital mortality. The longest follow-up mortality reported in the individual studies was extracted for the pooling analysis. Secondary outcomes included the ICU length of stay (LOS), hospital LOS, recovery of renal function, RRT dependence, duration of RRT, renal recovery time and mechanical ventilation time.

### Assessment of evidence quality

The quality of evidence of each study was assessed (Xiao-mei Yang and Jian Gao) according to the guidelines of the GRADE Working Group (http://www.gradeworkinggroup.org/index.html), using the GRADE profiler (version 3.6.1, http://ims.cochrane.org/revman/gradepro) and GRADE handbook to determine the quality of evidence and strength of recommendation.

### Statistical analysis

Meta-analysis was performed using the relative risks (RRs) for binary outcome and weighted mean difference (WMD) for continuous outcome measures. Alternatively, when there was no event in either groups during the follow-up, we used relative difference (RD), defined as the difference in the incidence rate of the early RRT group from that in the late RRT group. Data were pooled using a random effects model based on the inverse variance approach to give a more conservative estimate of the effect, allowing for any heterogeneity between studies. Statistical heterogeneity among studies was assessed by using the Q statistic and I^2^ statistics [14]. Meta-analyses were performed using RevMan 5.3 (Cochrane IMS, Oxford, UK, http://ims.cochrane.org/revman/download). All additional analyses were performed by using Stata/MP 12.1 (Stata Corp, College Station, TX, USA). A *P*-value < 0.05 was set as the threshold of statistical significance. Neither ethics board approval nor patient consent was required due to the nature of a systematic review.

Sensitivity analyses were conducted by excluding or subgrouping studies to reduce the potential confounding effects of patient population, RRT modality, study design, study sample size, duration of follow-up, urine output and creatinine. The log of the estimate of the study effect was set as the dependent variable in a general linear model, and the I2 and *P*-value were recalculated. Differences in the slopes of the linear regression models for the original and subgrouped data were used to predict the contributions of these potential confounding factors to the measured outcomes.

Risk of bias was assessed independently by 2 reviewers (Xiao-mei Yang and Guo-wei Tu) as recommended in the Cochrane Handbook, which includes 6 domains: selection bias, performance bias, detection bias, attrition bias, reporting bias, and other potential sources of bias. When there was insufficient information to allocate a high or low score, an “unclear” risk score was allocated. Disagreements in score allocations were resolved through group discussion. Publication bias was assessed using funnel plots, Begg’s test and Egger’s test [15], with *P* < 0.1 indicative of reporting biases.

## Results

### Study selection

A total of 1020 potentially relevant citations were identified and screened from databases. We retrieved 23 articles for full-text review, and 14 were excluded based on eligibility criteria. Nine RCTs fulfilled all criteria for the final meta-analysis (Fig. 1). All studies were written in English.

### Characteristics of the studies

The basic characteristics of the studies selected are shown in Table 1. A total of 1636 patients were enrolled. Of these studies, four of the studies were multi-center studies [12, 16–18], four were single-center studies [13, 19–21], and one was a two-center study [22]. Three studies examined only patients following cardiac surgery [17, 19, 20], whereas the remaining six studies were mixed with medical or surgical patients [12, 13, 16, 18, 21, 22]. The follow-up time reported in these studies ranged from 14 to 90 days.

**Table 1:** The Basic Characteristics of Studies Included in Meta-analysis

Author	Year	Country	Study Period	Study design	Population	Duration of Follow-up (days)	No. of Patients			Mean Age (year)		Male (%)	
							Total	Early	Late	Early	Late	Early	Late
Bouman	2002	Netherlands	1998–2000	Two center	Cardiac surgery/surgical/medical	28	106	70	36	EHV:68 ELV:70	LLV:67	EHV:60 ELV:57	LLV:61
Durmaz	2003	Turkey	1999–2001	Single-center	Cardiac Surgery	30	44	21	23	58	54	76	83
Sugahara	2004	Japan	1995–1997	Single-center	Cardiac Surgery	14	28	14	14	65	64	64	64
Payen	2009	France	1997–2000	Multicenter	Medical/surgical	28	76	37	39	58	59	73	69
Jamale	2013	India	2010–2012	Single-center	Medical	90	208	102	106	43	42	61	75
Combes	2015	France	2009–2012	Multicenter	Cardiac Surgery	90	224	112	112	61	58	79	80
Wald	2015	Canada	2012–2013	Multicenter	Medical/surgical	90	100	48	52	62	64	73	71
Gaudry	2016	France	2013–2016	Multicenter	Medical/surgical	60	619	311	308	65	67	67	64
Zarbock	2016	Germany	2013–2015	Single-center	Cardiac surgery/surgical	90	231	112	119	66	68	70	57

*Abbreviations*: *EHV* early high volume, *ELV* early low volume, *LLV* late low volume

The definition of early and late initiation of RRT for each specific study is outlined in Table 2. Five studies used urine output and/or serum creatinine or serum urea nitrogen or creatinine clearance for defining early and late RRT [18–22], two studies started early RRT when with diagnosis of severe sepsis or post-cardiac surgery shock [16, 17], and the latest two studies in 2016 used Kidney Disease: Improving Global Outcomes (KIDGO) stage 2 or stage 3 to define early RRT [12, 13]. In most of the studies, late RRT was defined as a classic indication, including azotemia, oliguria, pulmonary edema, hyperkalemia and metabolic acidosis. The individual studies defined early and late RRT by using variable cutoff values in serum creatinine or urine output.

**Table 2:** Definition of Early and Late RRT in Studies Included in the Meta-analysis

Author	Year	Early RRT Criteria	Late RRT Criteria
Bouman	2002	RRT within 12 h if urine output <30 ml/h, Cr clearance <20 ml/min, and mechanical ventilation	Urea > 40 mmol/L or K > 6.5 mmol/L or severe pulmonary edema
Durmaz	2003	Preoperative prophylactic RRT in all patients and postoperative sCr increased >10%	Postoperative sCr increased >50% or urine output <400 ml/24 h
Sugahara	2004	Urine output <30 ml/h for 3 h or urine output <750 ml/day	Urine output <20 ml/h for 2 h or urine output <500 ml/day
Payen	2009	RRT for 96-h period within 24 h of diagnosis of severe sepsis	Classic indications for RRT
Jamale	2013	Serum urea nitrogen >70 mg/dL and/or creatinine >7 mg/dL	Classic indications for RRT or Uremic nausea and anorexia
Combes	2015	RRT within 24 h of diagnosis of post-cardiac surgery shock	Creatinine >4 mg/dL or preoperative creatinine × 3 or urine output <0.3 ml/kg/h /24 h or urea >36 mmol/L or life-threatening hyperkalemia
Wald	2015	sCr increased >200%, urine output <6 ml/kg within 12 h, or NGAL ≥ 400 ng/ml	K > 6.0 mmol/L or serum bicarbonate <10 mmol/L or pulmonary edema
Gaudry	2016	RRT within 6 h of diagnosis of KDIGO stage 3	K > 6.0 mmol/L or PH < 7.15 or pulmonary edema or blood urea nitrogen >112 mg/dL or oliguria >72 h
Zarbock	2016	RRT within 8 h of diagnosis of KDIGO stage 2	RRT within 12 h of KDIGO stage 3 or no RRT

*Abbreviations*: *RRT* renal replacement therapy, *Cr* creatinine, *sCr* serum creatinine, *K* potassium, *KDIGO* kidney disease: improving global outcomes, *NGAL* neutrophil gelatinase-associated lipocalin

The modality of RRT varied significantly among the individual studies (Table 3). The modality of continuous vena-venous hemofiltration (CVVH) was used in five studies [13, 16, 17, 20, 22] and intermittent hemodialysis (IHD) was used in two studies [19, 21]. In the remaining two studies, some of the patients received CVVH modality, and the others received IHD or sustained low-efficiency dialysis (SLED) modality [12, 18]. At the time of initiation of RRT, the early group had serum creatinine ranging from 1.7 to 7.4 mg/dL and urine output from 400 to 1543 ml/day while the late group had serum creatinine ranging from 1.8 to 10.4 mg/dL and urine output ranging from 150 to 1491 ml/day (Table 3). Several studies reported illness severity measured by APACHE II score and SOFA score before RRT initiation. The APACHE II score ranged from 19 to 30.6 in the early group and 18 to 32.7 in the late group, whereas the SOFA score ranged from 7.6 to 15.6 in the early group and 8.2 to 16 in the late group (Table 3).

**Table 3:** The Characteristics at the time of renal replacement therapy initiation in studies included in Meta-analysis

Author	Modality	Creatinine (mg/d L)		Urine output (ml/24 h)		APACHE II score		SOFA score	
		Early	Late	Early	Late	Early	Late	Early	Late
Bouman	CVVH	NR	NR	NR	NR	EHV:23.5 (8.4) ELV:21.7 (5.5)	LLV:23.6 (8.3)	EHV:10.3 (2.8) ELV:10.1 (2.2)	LLV:10.6 (1.9)
Durmaz	IHD	3.1 (1.0)	4.3 (1.1)	NR	NR	NR	NR	NR	NR
Sugahara	CVVH	2.9 (0.2)	3.0 (0.2)	29 (1)^a^	18 (1)^a^	19 (2)	18 (3)	NR	NR
Payen	CVVH	2.1 (1.1)	2.1 (1.3)	1543 (209)	1491 (242)	NR	NR	NR	NR
Jamale	IHD	7.4 (5.3)	10.4 (3.3)	429 (388)	376 (350)	NR	NR	7.6 (3.3)	8.2 (3.1)
Combes	CVVH	1.7 (0.9)	1.8 (0.9)	NR	NR	NR	NR	11.5 (2.8)	12 (2.9)
Wald	IHD/CVVH/SLED	3.7 (1.4)	4.6 (2.2)	400 (211–568)	265 (80–755)	NR	NR	12 (3.3)	11.9 (2)
Gaudry	IHD/CVVH	3.3 (1.4)	5.3 (2.3)	NR	150 (50–600)	NR	NR	10.9 (3.2)	10.8 (3.1)
Zarbock	CVVH	1.9 (0.6)	2.4 (1)	445 (175–807.5)	270 (112.5–670)	30.6 (7.5)	32.7 (8.8)	15.6 (2.3)	16 (2.3)

*Abbreviations*: *NR* not reported, *APACHE* acute physiology and chronic health evaluation, *SOFA* sequential organ failure assessment, *EHV* early high volume, *ELV* early low volume, *LLV* late low volume, *CVVH* continuous venovenous hemofiltration, *IHD* intermittent hemodialysis, *SLED* sustained low-efficiency dialysis

^a^Sugahar reported urine output by ml/h

### Primary outcome

**Fig. 2:**
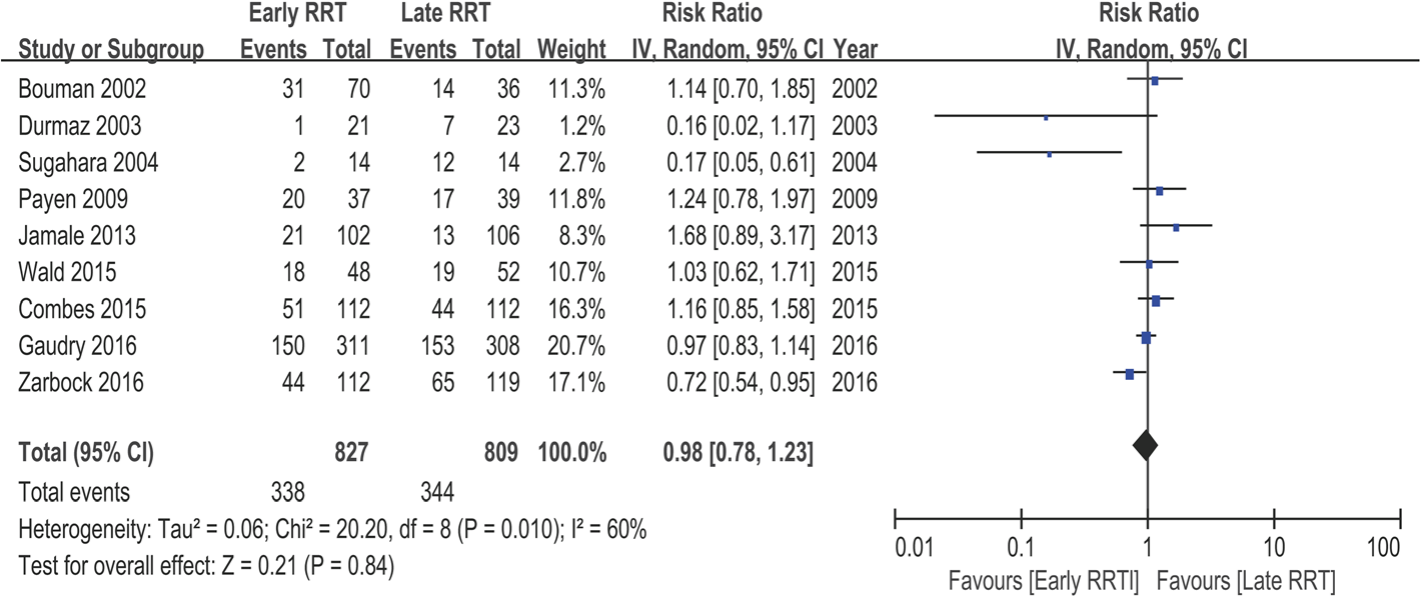
Forest plot for mortality of 9 studies

The definition of mortality reported in these studies differed considerably, including 14-day mortality [20], 28-day mortality [12, 13, 16, 22], 30-day mortality [17], 60-day mortality [12, 13, 17], 90-day mortality [13, 17, 18], ICU mortality [17, 18, 22], and in-hospital mortality [17–19, 21, 22]. We selected the longest follow-up mortality of each study as the primary end point. Figure 2 showed the mortalities of individual studies and pooled analysis. The total number of participants was 827 in the early group, with 338 deaths, and 809 in the late group, with 344 deaths. Pooled analysis of the studies indicated no mortality benefit with “early” RRT, with an RR of 0.98 (95% CI 0.78 to 1.23, *P* = 0.84). However, there was relatively high statistical heterogeneity with an I^2^ value of 60% (*P* = 0.01).

### Subgroup analysis

**Fig. 3:**
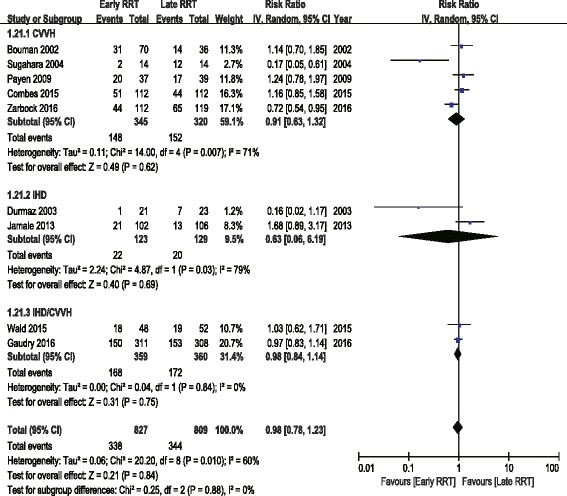
Forest plot for RRT modality

**Fig. 4:**
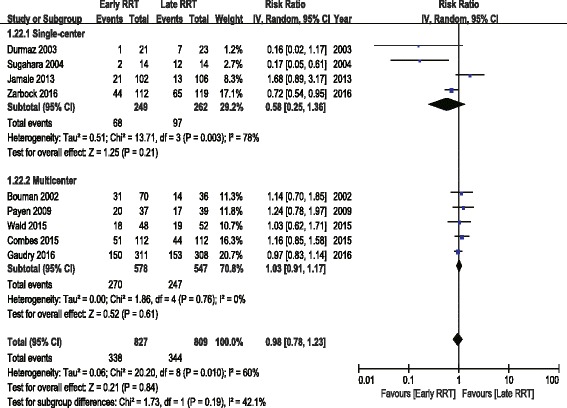
Forest plot for study centers

To explore the factors that may result in heterogeneity, several subgroup analyses were performed. Analyses grouped by patient population (post cardiac surgery versus multisystem), by RRT modality (CVVH versus IHD or IHD/CVVH), by study design (single center versus multicenter), by study sample size (≥100 versus <100), by duration of follow-up (≥100 days versus <100 days), by urine output (significant difference versus no significant difference between early and late RRT group), by creatinine (significant difference versus no significant difference between early and late RRT group), or by KDIGO classification of early RRT group (stage 1–2 versus stage 3) demonstrated no significant difference in the overall effect estimates. Meta-regression analyses showed no statistically significant association between RR and patient population, RRT modality, study design, study sample size, duration of follow-up, urine output, creatinine, and KDIGO classification (Table 4, Figs. 3 and 4).

**Table 4:** Subgroup Meta-analyses and Meta regression Analyses

Subgroup	No. of Studies	No. of Patients	Random-Effects Model RR (95% CI)	Test for Effect *P*	I^2^(%)	Test for Heterogeneity *P*	Meta regression *P*
All Studies	9	1636	0.98 (0.78, 1.23)	0.84	60	0.01	
Study Design							0.216
Single-center	4	511	0.58 (0.25, 1.36)	0.21	78	0.003	
Multicenter	5	1125	1.03 (0.91, 1.17)	0.61	0	0.75	
Sample Size							0.362
< 100	3	148	0.37 (0.07, 1.90)	0.23	82	0.004	
≥ 100	6	1488	1.00 (0.83, 1.21)	0.98	44	0.11	
Patient Population							
Multisystem	6	1340	1.00 (0.82, 1.22)	0.99	42	0.120	
Post cardiac surgery	3	296	0.37 (0.07, 1.81)	0.22	83	0.003	
RRT Modality							0.838
CVVH	5	665	0.91 (0.63, 1.32)	0.62	71	0.007	
IHD	2	252	0.63 (0.06, 6.19)	0.69	79	0.03	
CVVH/IHD	2	719	0.98 (0.84, 1.14)	0.75	0	0.84	
Days of Follow up							0.681
< 60	4	254	0.65 (0.30, 1.43)	0.29	74	0.009	
≥ 60	5	1382	0.99 (0.80, 1.23)	0.95	53	0.07	
Creatinine Difference							0.632
Significant	4	583	0.92 (0.55, 1.53)	0.74	67	0.03	
Nonsignificant	5	1053	1.02 (0.78, 1.33)	0.87	57	0.05	
UO Difference							0.079
Significant	2	259	0.40 (0.10, 1.63)	0.20	78	0.03	
Nonsignificant	5	1227	1.05 (0.92, 1.19)	0.48	0	0.42	
KDIGO							0.334
stage 1–2	5	627	0.74 (0.47, 1.17)	0.20	72	0.01	
stage 3	3	933	1.08 (0.84, 1.40)	0.54	31	0.23	

*Abbreviations*: *RRT* renal replacement therapy, *EHV* early high volume, *ELV* early low volume, *LLV* late low volume, *CVVH* continuous venovenous hemoflitration, *IHD* intermittent hemodialysis, *UO* urinary output, *KDIGO* kidney disease: improving global outcomes

### Secondary outcomes

**Fig. 5:**
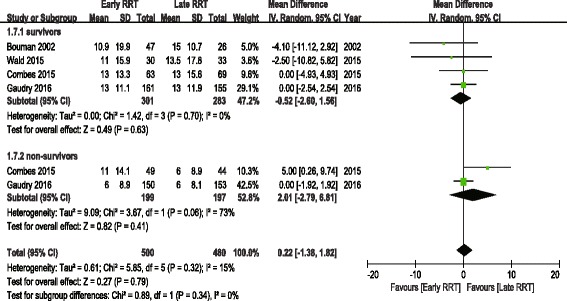
Forest plot for ICU Length of stay

**Fig. 6:**
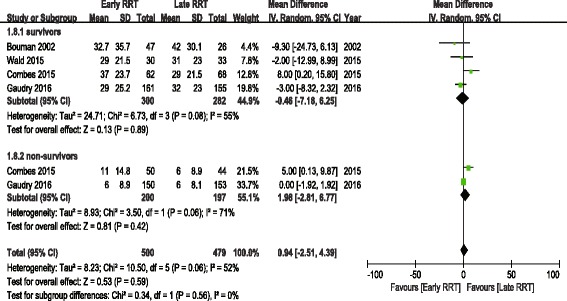
Forest plot for hospital length of stay

**Fig. 7:**
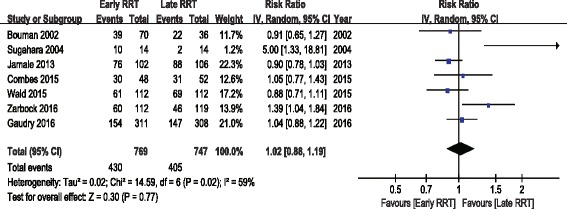
Forest plot for renal function recovery

**Fig. 8:**
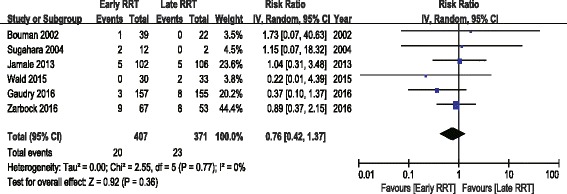
Forest plot for renal replacement therapy dependence

**Fig. 9:**
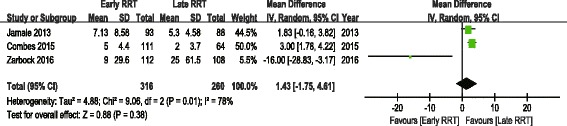
Forest plot for duration of renal replacement therapy

**Fig. 10:**
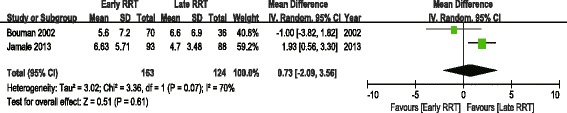
Forest plot for renal recovery time

**Fig. 11:**
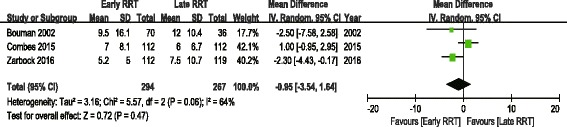
Forest plot for mechanical ventilation time

The analysis of secondary outcomes included the ICU LOS, hospital LOS, recovery of renal function, RRT dependence, duration of RRT, renal recovery time and mechanical ventilation time. Due to the variability in the reporting of ICU LOS and hospital LOS, we performed a pooled analysis for subgroups, including ICU LOS/hospital LOS in survivors and nonsurvivors. There was no significant difference in ICU LOS and heterogeneity between early and late RRT group for survivors or nonsurvivors (Fig. 5). A similar subgroup analysis performed on hospital LOS also showed no significant difference (Fig. 6). Seven of the nine studies reported recovery of renal function and RRT dependence. Pooled analysis also demonstrated no significant change in renal function recovery (RR 1.02, 95% CI 0.88 to 1.19, I^2^ = 59%; Fig. 7) or RRT dependence (RR 0.76, 95% CI 0.42 to 1.37, I^2^ = 0%; Fig. 8) between the early and late RRT groups. Three studies reported the duration of RRT [13, 17, 21], two studies reported renal recovery time [21, 22] and three studies reported mechanical ventilation time [13, 17, 22]. Pooled analysis of these studies showed no significant increase in duration of RRT in early RRT (Mean difference 1.43, 95% CI -1.75 to 4.61, I^2^ = 78%; Fig. 9), renal recovery time (Mean difference 0.73, 95% CI -2.09 to 3.56, I^2^ = 70%; Fig. 10) or mechanical ventilation time (Mean difference − 0.95, 95% CI -3.54 to 1.64, I^2^ = 64%; Fig. 11).

### Assessment of evidence quality

One critical outcome (the overall mortality of patients) and 7 important outcomes, including ICU LOS of survivors, hospital LOS of survivors, renal function recovery, renal recovery time, duration of RRT, RRT dependence and mechanical ventilation time, were evaluated using the GRADE system. The level of evidence quality was moderate for overall mortality and low for other secondary outcomes (Table 5).

**Table 5:** Rating the Quality of Evidences by GRADE

Outcomes	Studies	No. of Participants		Effect		Quality	Importance	Recommendation grade
		Early RRT	Late RRT	RR/RD (95% CI)	Absolute			
Overall mortality	9	338/827 (40.9%)	344/809 (42.5%)	RR 0.98 (0.78 to 1.23)	9 fewer per 1000 (from 94 fewer to 98 more)	⊕ ⊕ ⊕Ο Moderate	Critical	Weak
ICU LOS of survivors	4	301	283	-	MD 0.52 lower (2.6 lower to 1.56 higher)	⊕ ⊕ ΟΟ low	Important	Weak
Hospital LOS of survivors	4	300	282	-	MD 0.46 lower (7.18 lower to 6.25 higher)	⊕ ⊕ ΟΟ low	Important	Weak
Renal function recovery	7	430/769 (55.9%)	405/747 (54.2%)	RR 1.02 (0.88 to 1.19)	11 more per 1000 (from 65 fewer to 103 more)	⊕ ⊕ ΟΟ low	Important	Weak
Renal recovery time	2	163	124	-	MD 0.73 higher (2.09 lower to 3.56 higher)	⊕ ⊕ ΟΟ low	Important	Weak
Duration of RRT	3	316	260	-	MD 1.43 higher (1.75 lower to 4.61 higher)	⊕ ⊕ ΟΟ low	Important	Weak
RRT dependence	7	19/447 (4.3%)	23/427 (5.4%)	RR 0.76 (0.42 to 1.37)	13 fewer per 1000 (from 31 fewer to 20 more)	⊕ ⊕ ΟΟ low	Important	Weak
Mechanical ventilation time	3	294	267	-	MD 0.95 lower (3.54 lower to 1.64 higher)	⊕ ⊕ ΟΟ low	Important	Weak

*Abbreviations*: *RRT* renal replacement therapy, *ICU* intensive care unit, *LOS* length of stay, *GRADE* Grading of Recommendations Assessment, Development, and Evaluation, *No* number, *CI* confidence interval, *RD* relative difference, *RR* risk ratio, *MD* mean difference

### RRT-related complications

Several RRT-related complications were reported in the studies, including hemorrhage, thrombocytopenia, thrombosis, hypokalemia, hypophosphatemia, hyperkalemia, RRT-associated arrhythmia, RRT-associated seizure, hypothermia, catheter infection and hypotension during RRT (Table 6). We found no significant differences in complications between the early and late RRT groups, with no heterogeneity between trials, except for hypophosphatemia (I^2^ = 92%; *P <* 0.0001).

**Table 6:** RRT-related Complications of Studies Included in Meta-analysis

Outcomes	Study No.	No of patients	Risk Difference (95% CI)	*P* for Effect	I^2^	*P* for Model
hemorrhage	6	1458	0.00(−0.02, 0.01)	0.89	0%	0.73
thrombocytopenia	3	949	0.05(−0.04, 0.14)	0.25	60%	0.08
thrombosis	2	700	−0.01(−0.04, 0.02)	0.40	0%	0.58
hypokalemia	3	924	0.00(−0.08, 0.07)	0.89	61%	0.08
hypophosphatemia	3	924	0.11(−0.09, 0.31)	0.28	92%	<0.0001
hyperkalemia	2	843	−0.01(−0.04, 0.02)	0.41	0%	0.56
RRT-associated arrhythmia	3	920	−0.02(−0.08, 0.04)	0.48	58%	0.09
RRT-associated seizure	3	525	0.00(−0.01, 0.01)	0.85	0%	0.55
hypothermia	2	843	0.01(−0.01, 0.04)	0.28	0%	0.62
catheter infection	4	1014	0.01(−0.02, 0.04)	0.36	31%	0.23
hypotension during RRT	3	652	0.02(−0.02, 0.06)	0.37	37%	0.21

*Abbreviations*: *RRT* renal replacement therapy

### Risk of bias and publication bias

**Fig. 12:**
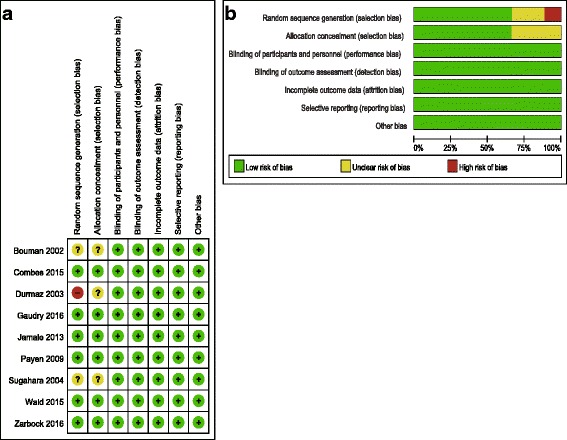
**a** Risk of bias summary: review authors’ judgments about each risk of bias item for each included study. **b** Risk of bias graph: review authors’ judgments about each risk of bias item presented as percentages across all included studies

**Fig. 13:**
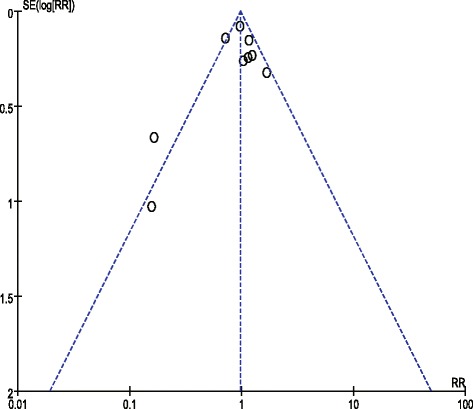
Assessment of publication bias using a funnel plot

Risk of bias is shown in Fig. 12. Of the nine studies, two studies did not report random sequence generation, and three studies did not report allocation concealment and were thus considered to have an unclear risk of bias. Overall, the included RCTs had a low risk of bias. The funnel plot was symmetrical (Fig. 13), which indicated the absence of publication bias between the trials included in our meta-analysis (*P* = 0.832, Egger’s test; *P* = 0.917, Begg’s test).

## Discussion

Although the need to initiate RRT is unequivocal in AKI patients with traditional indications, the advantages of commencing RRT in the absence of life-threatening complications are still under controversial [18]. Despite numerous low-quality studies in this field, the definitive conclusion is still yet to be elucidated. There is insufficient evidence to suggest that early initiation of RRT is related to reduced mortality or other patient-centered outcomes in critically ill patients. It should be mentioned that the spontaneous recovery of renal function may occur in certain patients with AKI. In the AKIKI study, up to 49% of the patients in the delayed-strategy group avoided receiving RRT [12]. However, we cannot accurately predict the needs for RRT or opportunity of renal recovery in critically ill patients in the retrospective studies. One may even argue that patients in the early RRT group might have less severe conditions or more opportunity to have a spontaneous recovery of renal function, both of which might account for the improved outcomes. Therefore, the optimal timing of initiation of RRT cannot be accurately gained from retrospective studies. Four meta-analyses indicated that “early” initiation of RRT might reduce the mortality of patients with AKI compared with “late” RRT, but most of the enrolled trials were retrospective cohort studies which could affect the facticity of the final conclusion [9, 11, 23, 24].

We enrolled 9 RCTs with a total of 1636 patients in this meta-analysis and found that “early” RRT had no beneficial effect on mortality of patients with AKI compared with “late” RRT. Furthermore, pooled analysis of these studies also showed no significant benefit of early RRT in renal function recovery, renal recovery time, RRT dependence, duration of RRT or mechanical ventilation time. It has been known that there are many differences between post-cardiac surgery patients and those with noncardiac surgery, especially on the perioperative hemodynamic management. However, subgroup analysis of the studies concerning the patients post cardiac surgery or those with noncardiac surgery did not reveal a survival benefit of early RRT intervention. In addition, the conclusion remained the same, regardless of whether early was defined by AKI stages according to the KDIGO classification or on the basis of urine output or serum creatinine.

One highlight of this meta-analysis was that we included two new large RCTs published recently [12, 13], which made our results more convincing. Second, RRT-related complications were evaluated in the meta-analysis, and no significant differences in complications between the early and late RRT groups were found. Third, survivors and nonsurvivors were analyzed separately in the secondary outcome analysis, and the same conclusion was reached. Fourth, this meta-analysis was performed under the requirements of Preferred Reporting Items for Systematic reviews and Meta-Analyses (PRISMA), which enhances the reliability of the conclusion. The quality of evidence and strength of recommendations were rated according to the guidelines of the GRADE Working Group.

However, facing the results we got, we could not confirm the conclusion definitively. There was relatively high heterogeneity with an I^2^ value of 60%, which might be explained by the significant variation in study design, population characteristics, baseline AKI severity, initiation time of RRT, modality used, and duration of follow-up among studies. The most fundamental differences among the trials were the huge differences concerning the timing of RRT initiation among studies. Urine output, serum creatinine, serum urea nitrogen and AKI stages were not used unified in the individual studies to define the early and late RRT strategies. In extreme cases, patients in the early RRT group in one study might be enrolled as late RRT in other studies. The high heterogeneity of definitions of “early” and “late” RRT between RCTs precluded the establishment of definitive conclusions. Second, most studies enrolled AKI patients with a mixed population; whereas the optimal timing of RRT initiation might be associated with the primary diseases. Moreover, the severity of the primary disease, presence of comorbid conditions, complications after surgery and fluid balance before RRT initiation could also be the possible confounders related to study outcome. Third, although the included 9 studies were all RCTs, the quality of many of them was not very high. There was a trend of publication bias towards a survival benefit from early initiation of RRT in studies with small sample sizes. In addition, different randomized methods were adopted in the individual studies, some of which were not sufficiently rigorous.

Additionally, we cannot omit the progression of the critical care medicine during the past decade. In the study conducted in patients with acute renal failure following coronary bypass surgery in 2004, the mortality was as high as 86% in the “late” group [20]. However, the HEROICS study, conducted in post-cardiac surgery shock patients in 2015, showed that the 30-day mortality of the “early” and “late” group was only 36% [17]. We found that 5/6 of the RCTs published over the past decade failed to prove the benefit of early initiation of RRT. Great progress in hemodynamic monitoring, mechanical ventilation, nutrition support and even RRT technology development has been achieved in critical care medicine in the past decade. This may also partially explain the negative results of large RCTs in recent years regarding the early goal-directed therapy in the treatment of severe sepsis and septic shock [25–27]. Therefore, studies published before 1985 were excluded in this meta-analysis.

Based on these limitations, there is no established evidence of the association between timing of RRT initiation and outcomes. Due to the relatively high heterogeneity among enrolled studies, the conclusion of the meta-analysis should be interpreted with great caution. Although we could not reach the definite conclusion in our meta-analysis, we raise several suggestions for upcoming studies: (1) enrolled patients might be in a specified population, such as sepsis or post cardiovascular surgery, avoiding mixed populations; (2) using a unified definition of the timing of early and late RRT could facilitate reaching reliable conclusions; and (3) the endpoint outcome of studies and choice of modalities of RRT should also be uniform if possible.

## Conclusions

Our meta-analysis revealed that the “early” initiation of RRT in critically ill patients did not result in a reduced mortality. A pooled analysis of secondary outcomes showed no significant difference in ICU LOS or hospital LOS between early and late RRT group for survivors or nonsurvivors. A pooled analysis also demonstrated no significant change in renal function recovery, RRT dependence, renal recovery time or mechanical ventilation time. No significant differences in complications between the early and late RRT groups were found. Due to the relatively high heterogeneity among enrolled studies in this meta-analysis, the conclusion of the meta-analysis should be interpreted with great caution. More well-designed and large-scale trials are expected to confirm the result of this meta-analysis.

## Additional file

Additional file 1: Table S1. Search strategy terms and results. (RTF 81 kb)

## Abbreviations

AKI: Acute kidney injury
CRRT: Continues renal replacement therapy
CVVH: Continuous veno-venous hemofiltration
ICU: Intensive care unit
IHD: Intermittent hemodialysis
LOS: Length of stay
RCTs: Randomized controlled trials
RRT: Renal replacement therapy
