# Supplementary material for: A comparison of early versus late initiation of renal replacement therapy for acute kidney injury in critically ill patients: an updated systematic review and meta-analysis of randomized controlled trials
Source: BMC Nephrol. 2017 Aug 7;18:264. doi: 10.1186/s12882-017-0667-6 (PMC5547509; doi:10.1186/s12882-017-0667-6)
Supplement: Additional file 1: Table S1. — Search strategy terms and results. (RTF 81 kb) [file 12882_2017_667_MOESM1_ESM.rtf]

Additional file 1

Table S1. Search strategy terms and results

PUBMED, searched from January 1, 1985 to June 30, 2016, clinical trial, humans	
1	Acute kidney injury [MeSH majr] OR “acute kidney” [ti/ab] OR “acute renal” [ti/ab]	1546	
2	Renal replacement therapy [MeSH majr] OR renal replacement[ti] OR hemodialysis [ti] OR hemofiltration [ti]OR dialysis [ti] OR dialyzed [ti] OR dialyzing [ti] 	10529	
3	Time to treatment [MeSH] OR Time factors [MeSH] OR time OR timing [ti/ab] OR initiation [ti/ab] OR start [ti/ab] OR accelerate [ti/ab] OR accelerated [ti/ab] OR accelerating [ti/ab] OR acceleration [ti/ab] OR early [ti/ab] OR earlier [ti/ab] OR late [ti/ab]	238982	
4	Combine #1 AND # 2 AND # 3	230	
5	Filters: English	217	


EMBASE, searched from January 1, 1985 to June 30, 2016, clinical trial, humans	
1	Acute kidney injury OR acute kidney OR acute renal	4624	
2	Renal replacement therapy OR renal replacement OR hemodialysis OR hemofiltration OR dialysis OR dialyzed OR dialyzing	13149	
3	Time to treatment OR time OR timing OR initiation OR start OR accelerate OR accelerated OR accelerating OR acceleration OR early OR earlier OR late	289329	
4	Combine #1 AND # 2 AND # 3 	582	
5	Limit to: English	559	


Cochrane, searched from 1985 to 2016, trials	
1	Acute kidney injury OR acute kidney OR acute renal	5810	
2	Renal replacement therapy OR renal replacement OR hemodialysis OR hemofiltration OR dialysis OR dialyzed OR dialyzing	9950	
3	Time to treatment OR time OR timing OR initiation OR start OR accelerate OR accelerated OR accelerating OR acceleration OR early OR earlier OR late	289509	
4	Combine #1 AND # 2 AND # 3 	593	
